# Viral Interference between Respiratory Viruses

**DOI:** 10.3201/eid2802.211727

**Published:** 2022-02

**Authors:** Jocelyne Piret, Guy Boivin

**Affiliations:** Centre de Recherche du Centre Hospitalier Universitaire de Québec‒Université Laval, Quebec City, Quebec, Canada

**Keywords:** viral interference, viruses, respiratory viruses, respiratory infections, influenza virus, respiratory syncytial virus, human rhinovirus, human metapneumovirus, severe acute respiratory syndrome coronavirus 2, SARS-CoV-2, coronavirus disease, COVID-19, zoonoses, innate immunity, interferon

## Abstract

Multiple respiratory viruses can concurrently or sequentially infect the respiratory tract and lead to virus‒virus interactions. Infection by a first virus could enhance or reduce infection and replication of a second virus, resulting in positive (additive or synergistic) or negative (antagonistic) interaction. The concept of viral interference has been demonstrated at the cellular, host, and population levels. The mechanisms involved in viral interference have been evaluated in differentiated airway epithelial cells and in animal models susceptible to the respiratory viruses of interest. A likely mechanism is the interferon response that could confer a temporary nonspecific immunity to the host. During the coronavirus disease pandemic, nonpharmacologic interventions have prevented the circulation of most respiratory viruses. Once the sanitary restrictions are lifted, circulation of seasonal respiratory viruses is expected to resume and will offer the opportunity to study their interactions, notably with severe acute respiratory syndrome coronavirus 2.

Several respiratory viruses can circulate during the same period and can concurrently or sequentially infect the respiratory tract, leading to virus‒virus interactions. At the host level, the course of infection of 1 virus might be influenced by prior or concurrent infection by another virus. Infection by a first virus could enhance or reduce infection and replication of a second virus, resulting in positive (additive or synergistic) or negative (antagonistic) interaction.

Positive virus‒virus interaction corresponds to a co-infection that might result in an increased disease severity and pathogenesis (e.g., severe acute respiratory syndrome coronavirus 2 [SARS-CoV-2] and influenza A[H1N1]pdm09 virus) (*1*). Negative virus‒virus interaction can be homologous or heterologous depending on whether the 2 viruses belong to the same family or to different serotypes or families. Homologous virus‒virus interaction implies that cross-reactive immunity against a first virus prevents infection with a second virus (e.g., among different influenza subtypes or lineages) (*2*). Heterologous viral interference relies on induction of a nonspecific innate immune response by a first virus that reduces or prevents infection and replication of a second virus (e.g., influenza A virus [IAV] and respiratory syncytial virus [RSV]) (*3*). The type of virus‒virus interaction (negative or positive) is probably dependent on the respiratory viruses involved, the timing of each infection, and the interplay between the response of the host to each virus. In this perspective, we focus more specifically on viral interference.

## Mechanisms of Negative and Positive Virus‒Virus Interactions

The more probable mechanism of negative viral interactions relies on the induction of a transient innate immunity by the interfering virus. Structural components of viruses are sensed by pattern recognition receptors in epithelial and immune cells (Figure) (*4*). This recognition triggers the expression of interferon (IFN)‒stimulated genes (ISGs) and type I (i.e., IFN-α/β) and type III (i.e., IFN-λ) IFNs. The IFN-α/β receptor is expressed on most cell types, whereas the IFN-λ receptor is predominantly present on epithelial cells of the gastrointestinal and respiratory tracts. Secreted IFNs bind to receptors present at the surface of infected and neighboring cells to amplify the expression of ISGs. This process leads to an antiviral defense program consisting in the production of effectors that directly inhibit viral replication, as well as cytokines and chemokines.

**Figure:**
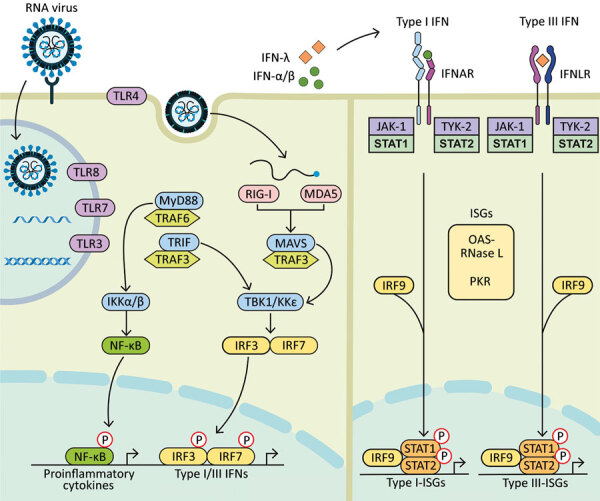
Diagram showing how components of RNA viruses are recognized by TLRs located at the plasma membrane (TLR4, viral glycoprotein sensing) and in the endosomal compartment (TLR3, double-stranded RNA sensing; TLR7 and TLR8, both single-stranded RNA sensing). Virus replication intermediates and replicated genomes are also recognized by cytosolic RNA sensors, RIG-I, and MDA5. Downstream adaptor proteins, MyD88 for TLR4, TLR7, and TLR8; TRIF for TLR3 and TLR4, and MAVS (for MDA5 and RIG-I) are activated. These activations trigger signaling cascades through TRAF3 and TRAF6; TBK1; and IKKα, IKKβ, and IKKε, which leads to phosphorylation and nuclear translocation of NF-κB, IRF3, and IRF7. These changes result in production of proinflammatory cytokines and type I and type III IFNs. Secreted IFN-α/β and IFN-λ bind to their specific receptors (IFNAR and IFNLR) in infected and neighboring cells. Activation of JAK-1 and TYK-2 leads to phosphorylation of STAT1 and STAT2. After translocation in the nucleus, phosphorylated STAT1 and STAT2 form a complex with IRF9 to induce expression of ISGs, such as OAS-RNase L and PKR, and establishment of an antiviral program. IFN, interferon; IFNAR, IFN-α/β receptor; IFNLR, interferon-λ receptor; IKK, inhibitor of nuclear factor-κB kinase; ISGs, IFN-stimulated genes; IRF, IFN regulatory factor; JAK-1, Janus kinase 1; MAVS, mitochondrial antiviral signaling protein; MDA5, melanoma differentiation-associated gene 5; MyD88, myeloid differentiation factor 88; NF-κB, nuclear factor-κB; OAS, 2′-5′ oligoadenylate synthetase; P, phosphorylated protein; PKR, protein kinase receptor; RNase L, latent endoribonuclease; RIG-I, retinoic acid‒inducible gene I; STAT, signal transducer and activator of transcription; TBK 1, TANK binding kinase 1; TLRs, Toll-like receptors; TRAF, tumor necrosis factor receptor-associated factor; TRIF, TIR-domain-containing adapter-inducing IFN-β; TYK-2, tyrosine kinase 2.

Induction of ISGs by a first virus might limit infection and replication of a second virus, especially if they show a differential ability to induce an IFN response or different degrees of susceptibility to immune mediators. To evade the immune system, respiratory viruses have developed a series of mechanisms that counteract the induction and antiviral action of IFNs, which might influence the type of virus‒virus interactions. Influenza viruses and SARS-CoV-2 have developed a broader range of multifaceted strategies to escape IFN induction and signaling than RSV, human metapneumovirus (HMPV) and human rhinovirus (HRV) (Table 1).

**Table 1:** Evasion mechanisms of human respiratory viruses to type I interferon*

Virus	Viral proteins interfering with interferon induction and signaling	Reference
Human rhinovirus	IFN induction: VPg interferes with viral RNA recognition by RNA sensors; 2A protease reduces cap-dependent translation of cellular mRNA; 2A and 3C proteases cleave MAVS. IFN signaling: 3C protease inhibits activation of antiviral protein complexes.	(*5*)
Human metapneumovirus	IFN induction: G interferes with TLR4 signaling; SH inhibits NF-κB signaling; M2.2 protein interferes with MAVS and inhibits IRF7 phosphorylation. IFN signaling: SH prevents STAT1 phosphorylation.	(*6*)
Respiratory syncytial virus	IFN induction: NS1 inhibits IRF3 phosphorylation, inhibits TRIM25-mediated RIG-I ubiquitination; NS2 binds to RIG-I and reduces IRF3 activation; G reduces IFN-λ production. IFN signaling: NS1 promotes OASL degradation and inhibits IFNAR1 expression; NS1 and NS2 induce STAT2 degradation.	(*5*)
Influenza virus	IFN induction: NS1 interferes with viral RNA sensing by TLR and RIG-I, binds to viral RNA and reduces RIG-I activation, inhibits TRIM25-mediated RIG-I ubiquitination and prevents the export of cellular mRNA to cytoplasm; PB1-F2 and PB2 interfere with MAVS; PA reduces IRF3 activation; M2 protein interacts with MAVS. IFN signaling: NS1 reduces PKR and OASL activation; HA induces IFNAR1 degradation; SOCS inhibits STAT2; NP and M2 protein interfere with PKR activation.	(*7*)
Severe acute respiratory syndrome coronavirus	IFN induction: NSP14 methylates capped RNA transcripts; NSP15 cleaves 5′-polyuridines from viral RNA; NSP16 and NSP10 methylate viral RNA cap; N protein inhibits TRIM25-mediated RIG-I ubiquitination; NSP3 deubiquitinates cellular substrates (possibly RIG-I) and inhibits IRF3 phosphorylation; ORF9b targets MAVS, TRAF3 and TRAF6 to degradation; M protein impedes TRAF3/TBK1/IKKε complex formation; ORF3b might target MAVS; NSP1 promotes cellular mRNA degradation and prevents host mRNA translation. IFN signaling: ORF3a promotes IFNAR1 degradation; NSP1 decreases STAT1 phosphorylation; ORF6 inhibits nuclear translocation of STAT1.	(*8*)

*G, glycoprotein; HA, hemagglutinin; IFN, interferon; IFNAR1, IFN-α/β receptor 1; IRF, IFN regulatory factor; M, matrix; MAVS, mitochondrial antiviral signaling protein; N, nucleocapsid; NP, nucleocapsid protein; NS, nonstructural; NSP, nonstructural protein, OASL, 2’-5′ oligoadenylate synthetase-ribonuclease L; ORF, open reading frame; PA, polymerase acidic; PB, polymerase basic; PKR, protein kinase receptor; RIG-I, retinoic acid‒inducible gene I; SH, viroporin protein; SOCS, suppressor of cytokine signaling; STAT, signal transducer and activator of transcription; TANK, TRAF family member‒associated NF-κB activator; TBK1, TANK binding kinase 1; TLR, Toll-like receptor; TRAF, tumor necrosis factor receptor‒associated factor; TRIM25, tripartite motif containing 25.

At the cellular level, blocking or reduction of cell surface receptors and competition for cellular resources and factors were suggested as mechanisms of negative virus‒virus interaction. For instance, the expression of neuraminidase in 293T cells infected with influenza A(H1N1) or A(H3N2) viruses can prevent a subsequent infection with retroviruses pseudotyped with a range of hemagglutinin molecules or a second IAV by removing sialic acid from the cell surface (*9*). Furthermore, replication of RSV was inhibited during co-infection with IAV in MDCK cells by competition for viral protein synthesis and budding from infected cells (*10*).

Other mechanisms leading to reduced or increased viral replication include the down-regulation or up-regulation of the gene promotor of a virus by a gene product of an interfering virus, but these mechanisms have not been yet demonstrated for respiratory viruses. Positive virus‒virus interaction could result from the formation of syncytia. For instance, the cell‒cell fusion activity of human parainfluenza virus type 2 was shown to enhance the growth of IAV in Vero cells (*11*). Co-infection could also increase disease severity through an overzealous production of IFNs or proinflammatory cytokines or through a reduced secretion of noninflammatory mediators, such as interleukin 10 (*12*).

## Viral Interference

The concept of viral interference was described by the research group of Voroshilova in the 1960s (*13*). This group developed live enterovirus vaccines (LEV) consisting of naturally attenuated enteroviruses for the prevention of enteric diseases that are caused by a large number of unrelated enteric pathogens, mainly in children. In addition to LEV’s protective effect on pathogenic enteroviruses, in particular polioviruses, oral administration of LEV in children decreased detection of several unrelated respiratory viruses, such as influenza virus, parainfluenza virus, RSV, HRV, and human adenovirus. This effect was suggestive of a phenomenon of viral interference that could be mediated through the IFN-inducing effect of LEV. During the 1968–1971 fall–winter seasons, large, controlled trials indicated that healthy adults immunized with LEV and oral polio vaccine showed a 2.6-fold and 3.8-fold decrease, respectively, in acute respiratory infections compared with adults who were not immunized (*14*). This study demonstrated that LEV and oral polio vaccine might confer protection against acute viral respiratory infections. However, the interest was dampened by the occurrence of rare cases of circulating vaccine-derived poliovirus (person-to-person transmission) and vaccine-associated paralytic poliomyelitis, a serious side effect.

## Advantages and Limitations of Ex Vivo and In Vivo Models

Three-dimensional models consisting of multiple differentiated nasal or bronchial epithelial cells that are polarized and share common characteristics with the human airway epithelium have been commonly used to characterize viral interference (*15*). The permeability and integrity of the reconstituted nasal or bronchial epithelia are ensured by the formation of tight junctions between epithelial cells. Differentiated human nasal or bronchial epithelia are cultured at the air‒liquid interface. These epithelia show active beating of cilia and are able to produce mucus. They can be infected by respiratory viruses and secrete IFNs and other immune mediators. Although these ex vivo models are limited by the absence of some immune cells that could contribute to viral interference, they are convenient tools to characterize the mechanisms of virus‒virus interaction at the mucosal level.

Animal models that are susceptible to several human respiratory viruses, such as ferrets and golden Syrian hamsters, are also valuable to evaluate the effects of concurrent and sequential viral infections on disease severity, immune response and the mechanisms of virus‒virus interaction at the host level (*16*). However, the immune response against human respiratory viruses and the mechanisms of immune evasion might differ between animal models and humans, which constitutes a potential limitation.

## Potential Interferences between Respiratory Viruses

A series of potential interferences between different respiratory viruses are demonstrated in epidemiologic studies and at the host level (Table 2). In some cases, the mechanisms involved in viral interference were investigated in differentiated human airway epithelial cells and in animal models.

**Table 2:** Potential viral interferences between respiratory viruses*

Interfering virus	Second virus	Observed effect in patients, animal models, and ex vivo systems	Results and statistical significance	Reference
pH1N1	H3N2	Prevents A(H3N2) shedding in ferret model	No H3N2 virus shedding	(*17*)
	IBV	Prevents or delays IBV shedding in ferret model	Peak delayed by 1.8 d (p = 0.014)	(*17*)
IAV	RSV	Reduced likelihood of co-detection in patients	OR 0.11 (95% CI 0.00–0.92)	(*18*)
		Reduced likelihood of co-detection in patients	OR 0.37 (95% CI 0.24–0.57)	(*19*)
		Prevents or delays RSV shedding in ferret model	Peak delayed by 2 d (p = 0.009)	(*3*)
RSV	HMPV	Reduced likelihood of co-detection in patients	OR 0.27 (95% CI 0.09–0.80)	(*19*)
		Reduces HMPV replication in HAEC model	By 1 or 2 log after 5 d (p<0.05)	(*20*)
HRV	IAV	Reduced likelihood of co-detection in patients	OR 0.06 (95% CI 0.01–0.24)	(*18*)
		Reduced likelihood of co-detection in patients	OR 0.08 (95% CI 0.02–0.30)	(*21*)
		Reduced likelihood of co-detection in patients	OR 0.15 (95% CI 0.04–0.53)	(*22*)
		Reduced likelihood of co-detection in patients	OR 0.16 (95% CI 0.09–0.28)	(*23*)
		Reduces IAV replication in HAEC model	>15-fold after 24 h (p = 0.0002)	(*23*)
RSV	HRV	Reduced infection rate with HRV in patients	8% vs. 14% (p<0.049)	(*24*)
		Reduced likelihood of co-infection in patients	OR 0.17 (95% CI 0.09–0.33)	(*18*)
		TCRI study	OR 0.30 (95% CI 0.22‒0.40)	(25)
		INSPIRE study	OR 0.18 (95% CI 0.11–0.28)	(*25*)
		MAKI trial	OR 0.34 (95% CI 0.16–0.72)	(*25*)
HRV	SARS-CoV-2	Reduces SARS-CoV-2 replication in HAEC model	By 3 log after 48 h (p = 0.006)	(*26*)
			By 3.5 log after 72 h (p<0.0001)	(*27*)

*HAEC, human airway epithelial cells; HMPV, human metapneumovirus; HRV, human rhinovirus; IAV, influenza A virus; IBV, influenza B virus; INSPIRE, Infant Susceptibility to Pulmonary Infections and Asthma Following RSV Exposure (in a region of the southeastern United States); MAKI, trial on the effects of RSV prophylaxis with palivuzimab in healthy preterm infants in the Netherlands; OR, odds ratio; RSV, respiratory syncytial virus; SARS-CoV-2, severe acute respiratory syndrome coronavirus 2; TCRI, Tennessee Children’s Respiratory Initiative.

## Influenza Virus Types and Subtypes

Influenza A(H1N1) virus reemerged during 1977 and cocirculated with seasonal influenza A(H3N2) before being replaced by the influenza A(H1N1)pdm09 pandemic virus. During the 1977–78 winter season in Japan, the percentage of children infected with H1N1 virus was lower for those infected shortly before with H3N2 virus than for persons who were not infected with H3N2 virus (59% vs. 91%; p<0.05) in 3 schools in which sequential outbreaks were observed (*28*). In a fourth school in which H3N2 and H1N1 virus outbreaks occurred concurrently, the rates of infection for children who had both viruses was lower than in the first 3 schools (2% vs. 21%, 23%, and 31%; p<0.05 for all). This study suggested that 2 mechanisms are at play in cross-subtype protection (i.e., antibody production during sequential outbreaks and viral interference during a mixed outbreak).

During the 2009–2011 influenza A(H1N1)pdm09 virus pandemic, several countries recorded distinct influenza epidemic peaks. During 2009, only pH1N1 virus circulated during the influenza season (weeks 23–36) in most temperate countries of the southern hemisphere. In contrast, a typical seasonal H3N2 peak (week 33) preceded the first pH1N1 wave (weeks 34–38) in South Africa (*29*). During the same year, a small seasonal H3N2 peak (week 34) occurred before the pH1N1 wave (weeks 44–54) and a subsequent influenza B virus (IBV) peak (week 4 of 2010) in Beijing (*30*). 

The temporal patterns of the different influenza epidemic peaks suggests a hierarchy between these viruses. The potential interference between influenza subtypes (pH1N1 and H3N2 and types (pH1N1 and IBV) has been evaluated in a ferret model (*17*). The disease outcome (i.e., shedding of co-infecting viruses in nasal wash specimens) varied with respect to the timing of the first and second infections. When the time interval was <3 days, co-infections occurred in almost all ferrets. Interferences between influenza virus types and subtypes were observed when sequential infections were attempted in an interval ranging from 3 to 7 days (Table 2). For this period, the authors suggested that innate immunity and intrinsic antiviral factors mediated by infection of ferrets with the interfering virus may prevent or delay infection and replication of the second virus (*17*). The pH1N1 virus was the most potent inducer of a protective immunity compared with IBV, but H3N2 virus was the less potent. In ferrets sequentially infected with 2 different IBV lineages, innate immunity and cross-reactive protection mediated by an IFN-γ response were involved (*2*).

## IAV and RSV

Surveillance of respiratory viral infections in Norway showed that RSV was less frequently detected during influenza epidemics, suggesting viral interference (*31*). An epidemiologic interference between influenza and RSV was also reported during different winter seasons in other countries (*32**,**33*). During 2002‒2017, it was estimated that RSV circulated an average of 6 weeks before IAV in Victoria, Australia (*19*). During the pH1N1 pandemic, the shift in influenza activity was associated with a change in seasonal RSV activity that further supports viral interference (*34**–**38*). Moreover, the probability of co-detecting both viruses was lower than expected from random associations; odds ratios (ORs) were <1 in 2 studies (Table 2), suggesting a negative interaction between IAV and RSV (*18**,**19*). In the ferret model, infection with pH1N1 virus prevented a subsequent infection with RSV for <7 days as assessed by viral shedding in nasal wash specimens (Table 2) (*3*). A first infection with RSV reduced the morbidity (i.e., duration of viral shedding and bodyweight loss) associated with a second challenge with pH1N1 virus, but all ferrets were co-infected. Infection of ferrets with pH1N1 virus induced a higher production of cytokines, chemokines, and immune mediators in the respiratory tract compared with RSV. However, both viruses induced only a low number of cross-reactive IFN-γ‒producing cells. These data suggest that innate immune mechanisms might be involved in interference between IAV and RSV.

## RSV and HMPV

RSV and HMPV co-circulate during winter and spring and can be co-detected in nasopharyngeal swab specimens of patients. Nevertheless, the type of interaction between these 2 pneumoviruses is controversial. A study reported that the likelihood of co-detecting RSV and HMPV in patients was reduced compared with expected values (OR 0.27, 95% CI 0.09–0.80), suggesting that a viral interference could occur (*19*). Differentiated human lung epithelial cells preinfected with RSV were less permissive to HMPV (Table 2), but the opposite was not detected (*20*). HMPV was more sensitive to IFN-α and IFN-λ than was RSV. IFN-α had a stronger antiviral effect on the 2 viruses compared with IFN-λ. The inhibition of HMPV replication by RSV was partially prevented in human lung adenocarcinoma A549 cells deficient for signal transducer and activator of transcription 1, suggesting that viral interference was partially mediated by the host innate immune response. Furthermore, inhibition of HMPV replication by RSV was also greatly reduced by antibodies against IFN-I and IFN-III.

## HRV and IAV

Many studies reported that the 2009 autumn epidemic of HRV might have delayed the circulation of pH1N1 in several countries in Europe (*39**–**41*). During 2014, a higher rate of HRV infections might have affected the subsequent influenza summer peak and even prevented the influenza epidemic in Hong Kong, China (*42*). Asynchronous epidemic peaks of HRV and IAV infections in adult patients were recorded during the 2017–2019 winter seasons at Yale‒New Haven Hospital (New Haven, CT, USA) (*23*). Furthermore, co-detection of HRV and IAV was lower than expected from random associations; ORs were <1 in several studies (Table 2), suggesting a negative virus‒virus interaction (*18**,**21**–**23*). Although mice do not support the complete replication process of HRV, its inoculation 2 days before IAV reduced the severity of influenza disease (i.e., clinical signs and bodyweight loss) and prevented deaths of animals (*43*). In contrast, HRV was less effective at protecting mice when given concomitantly with IAV. The protective effect of HRV was associated with an early but controlled pulmonary inflammatory response that enabled rapid clearance of IAV. Furthermore, infection of differentiated human airway epithelial cells with HRV protected against subsequent IAV or pH1N1 infection for up to 3 days (Table 2) (*23*). HRV infection induced expression of several ISGs, and blocking the IFN signaling pathways with BX795, an inhibitor of TANK binding kinase 1, restored pH1N1 virus replication.

## RSV and HRV 

Detection of RSV was associated with a reduced probability of co-detecting HRV in clinical specimens (OR 0.17, 95% CI 0.09–0.80), indicating a negative virus‒virus interaction (*18*). A negative interaction between RSV and HRV was consistently observed across diverse disease severity patterns, populations, seasons and geographic regions when analyzing 3 cohorts from the United States and the Netherlands (Table 2) (*25*). The rate of HRV infections was lower in children with recent RSV infection compared with children who were not infected (8% vs. 14%; p<0.049) (*24*). However, the median duration of symptoms was longer in children who were co-infected (that possibly occur outside of temporary immunity window) compared with children who had a single RSV infection (14 days vs. 11 days; p<0.02), suggesting an increased disease severity. Furthermore, HRV infections were more common in infants given immunoprophylaxis (palivizumab) against RSV than in infants who did not receive this drug (70.7% vs. 59.4%; OR 1.65, 95% CI 1.65–2.39) (*25*).

## HRV and SARS-CoV-2

In contrast to most respiratory viruses, HRV continued to circulate despite the mitigation measures put in place during the COVID-19 pandemic. HRV is a nonenveloped virus that is more resistant to hydroalcoholic disinfectant (*44*), and its transmission is not prevented by face masks (*45*). Studies showed that previous infection of human bronchial epithelial cells with HRV impairs replication of SARS-CoV-2 (Table 2), but the opposite was not detected (*26*). HRV triggers induction of several ISGs and blocks SARS-CoV-2 replication (*27*). Inhibition of ISG induction by BX795 abrogates the interference mediated by HRV and enhances the replication rate of SARS-CoV-2.

## Interactions between Influenza Virus and SARS-CoV-2

SARS-CoV-2 was shown to trigger a broader up-regulation of ISGs, cytokines and chemokines in the human nasal mucosa than pH1N1 virus (*46*). However, contrarily to influenza virus, SARS-CoV-2 fails to induce an early IFN-I and IFN-III response in human lung tissues, leading to a late and vigorous inflammatory response. Thus, the differential innate immune responses induced by SARS-CoV-2 and influenza virus in the upper and lower respiratory tracts might influence the type of virus–virus interactions, depending on which virus will infect first. Sequential infection of golden Syrian hamsters with pH1N1 and SARS-CoV-2 resulted in lower pulmonary SARS-CoV-2 load, suggesting a reduced replication in this tissue (*1*). In contrast, previous infection with SARS-CoV-2 did not affect pH1N1 load in the lungs compared with a single infection. Lung inflammatory damage and disease severity (i.e., clinical scores and bodyweight loss) were higher in animals infected with both viruses compared with a single virus. In this study, both viruses were inoculated into hamsters 24 hours apart, which might have been too short a time to induce interference. In ferrets first infected with influenza virus, there was a lag of 1–2 days before a nonspecific immune response was elicited and during which a co-infection with a second virus was likely to occur (*17*). Thereafter, the host innate immune response correlates with viral shedding in nasal wash specimens, which peaks at 2–3 days and persists for 5–6 days, corresponding to the window period when viral interference occurs. Thus, further studies are needed to clarify the interactions between SARS-CoV-2 and influenza viruses.

## Defective Viral Genomes, a Novel Therapeutic Option Based on Viral Interference

Defective viral genomes (DVGs) are produced during replication of RNA viruses and are believed to play a role in adaptation, viral escape, and persistence (*47*). DVGs have severe genomic truncations/modifications and require a full-length helper virus to replicate. DVGs are packaged, forming virus particles that are biochemically and morphologically similar to standard virus. DVGs might hamper the cytopathic effects induced by a wild-type virus. DVGs also rapidly produce cytopathic effects and interfere with replication of other co-infecting homologous or heterologous viruses. DVGs resulting from influenza virus replication can mediate homologous interference by competing with viral genomes for replication or packaging. DVGs might also mediate heterologous interference through production of IFN-I and IFN-III.

The role of DVGs in viral interference is not clearly established, but it is suggested that they could be used as therapeutic interfering particles against respiratory virus infections. In this respect, a first infection of mice with influenza A–based defective interfering virus, which was derived by a single central deletion from the full-length genomic segment 1 of influenza virus isolate A/PR/8/34 (H1N1), prevented disease caused by a second infection with a heterologous IBV (*48*). Protection against IBV was partially alleviated in mice that did not express a functional type I IFN receptor. Furthermore, a first infection with influenza A‒based defective interfering virus also protected mice against a second infection with pneumonia virus, a genetically unrelated respiratory virus (*49*).

## Conclusions and Perspectives

Recent viral infections of the respiratory tract might induce a refractory period during which the host is less likely to be infected by another respiratory virus. This viral interference requires closely spaced virus co-exposures, implying that both viruses share common ecologic conditions (e.g., cold weather). Factors that could predict an interference between respiratory viruses include the capacity of the interfering virus to induce a rapid IFN response; the degree of susceptibility of the second virus to immune mediators; the extent to which the different viruses counteract the induction and antiviral effects of IFN; and the differential innate immune response patterns triggered by each viruses in the upper and lower respiratory tracts.

The duration of the refractory period at the host level has not been determined, but might correspond to the period of virus shedding and the associated transient innate immune response. Mathematical models that simulate the co-circulation of seasonal IAV and HRV confirmed that the temporary immunity provided by an IFN response might be sufficient to produce the asynchronous epidemic peaks recorded for these 2 viruses (*12*). At the population level, the concept of viral interference corresponds to an ecologic phenomenon in which the epidemic caused by one virus delays the start or advances the end of the epidemic caused by another virus. These episodes are difficult to demonstrate because the transmission dynamics of respiratory viruses might be influenced by social behaviors for different age groups. The contact rate between persons might also vary according to different periods of the year, such as during school opening and closing. Furthermore, a large proportion of respiratory infections are asymptomatic and do not require testing, thus, excluding this part of the population from studies. Environmental conditions such as temperature and humidity can be confounding factors for viral interference. Prospective epidemiologic studies enabling detection of multiple respiratory viruses in serial nasopharyngeal swab specimens of participants over several epidemic periods would enable demonstration of viral interference. The type of interaction between respiratory viruses producing distinct epidemic peaks should be then confirmed by evaluating their likelihood of co-detection in patients, as well as the mechanisms involved in ex vivo and in vivo models.

The reappearance of H1N1 virus during 1977 and the 2009–2011 pH1N1 pandemic offered the opportunity to study the epidemiologic interactions between the newly circulating virus and seasonal respiratory viruses in northern and southern hemispheres and thus strengthened the concept of viral interference. During the COVID-19 pandemic, nonpharmacologic interventions have prevented the circulation of most respiratory viruses. Therefore, their potential interactions with SARS-CoV-2 could not be determined in epidemiologic studies, except in some reports at the onset of the pandemic. A systematic review and meta-analysis showed that the most common respiratory viruses co-detected with SARS-CoV-2 were influenza viruses, RSV, and HRV (*50*). Once the sanitary restrictions are lifted, the circulation of seasonal respiratory viruses should resume and different types of interactions are expected to occur.

Mathematical modeling predicting the timing and magnitude of epidemics caused by SARS-CoV-2 and seasonal respiratory viruses might improve public health interventions to protect persons at risk for co-infection through introduction of nonpharmacologic measures, adjustment of vaccine schedules, or use of prophylactic agents. Finally, the interfering and immunostimulatory activities of DVGs make them attractive candidates for development of prophylactic broad-spectrum antiviral drugs or vaccine adjuvant, which would be based on the concept of viral interference (*47*).
